# Evaluate effect of 126 pre-processing methods on various artificial intelligence models accuracy versus normal mode to predict groundwater level (case study: Hamedan-Bahar Plain, Iran)

**DOI:** 10.1016/j.heliyon.2024.e29006

**Published:** 2024-04-02

**Authors:** Mohsen Saroughi, Ehsan Mirzania, Mohammed Achite, Okan Mert Katipoğlu, Nadhir Al-Ansari, Dinesh Kumar Vishwakarma, Il-Moon Chung, Maha Awjan Alreshidi, Krishna Kumar Yadav

**Affiliations:** a Department of Irrigation and Reclamation Engineering, Faculty of Agricultural Engineering and Technology, College of Agriculture and Natural Resources, University of Tehran, Karaj, Iran; b Department of Water Engineering, University of Tabriz, Tabriz, Iran; c Faculty of Nature and Life Sciences, Laboratory of Water and Environment, Hassiba Benbouali University of Chlef, Chlef, 02180, Algeria; d Department of Civil Engineering, Erzincan Binali Yıldırım University, Erzincan, Turkey; e Department of Civil, Environmental, and Natural Resources Engineering, Lulea University of Technology, 97187, Lulea, Sweden; f Department of Irrigation and Drainage Engineering, Govind Ballabh Pant University of Agriculture and Technology, Pantnagar, Udham Singh Nagar, Uttarakhand, 263145, India; g Department of Water Resources and River Research, Korea Institute of Civil Engineering and Building Technology, Goyang-si, 10223, Republic of Korea; h Department of Chemistry, University of Ha'il, Ha'il, 81441, Saudi Arabia; i Faculty of Science and Technology, Madhyanchal Professional University, Ratibad, Bhopal, 462044, India; j Environmental and Atmospheric Sciences Research Group, Scientific Research Center, Al-Ayen University, Thi-Qar, Nasiriyah, 64001, Iraq

**Keywords:** Artificial intelligence, Deep learning, Groundwater level, Hybrid algorithm, Machine learning

## Abstract

The estimation of groundwater levels is crucial and an important step in ensuring sustainable management of water resources. In this paper, selected piezometers of the Hamedan-Bahar plain located in west of Iran. The main objective of this study is to compare effect of various pre-processing methods on input data for different artificial intelligence (AI) models to predict groundwater levels (GWLs). The observed GWL, evaporation, precipitation, and temperature were used as input variables in the AI algorithms. Firstly, 126 method of data pre-processing was done by python programming which are classified into three classes: 1- statistical methods, 2- wavelet transform methods and 3- decomposition methods; later, various pre-processed data used by four types of widely used AI models with different kernels, which includes: Support Vector Machine (SVR), Artificial Neural Network (ANN), Long-Short Term memory (LSTM), and Pelican Optimization Algorithm (POA) - Artificial Neural Network (POA-ANN) are classified into three classes: 1- machine learning (SVR and ANN), 2- deep learning (LSTM) and 3- hybrid-ML (POA-ANN) models, to predict groundwater levels (GWLs). Akaike Information Criterion (AIC) were used to evaluate and validate the predictive accuracy of algorithms. According to the results, based on summation (train and test phases) of AIC value of 1778 models, average of AIC values for ML, DL, hybrid-ML classes, was decreased to −25.3%, −29.6% and −57.8%, respectively. Therefore, the results showed that all data pre-processing methods do not lead to improvement of prediction accuracy, and they should be selected very carefully by trial and error. In conclusion, wavelet-ANN model with daubechies 13 and 25 neurons (db13_ANN_25) is the best model to predict GWL that has −204.9 value for AIC which has grown by 5.23% (−194.7) compared to the state without any pre-processing method (ANN_Relu_25).

## 1 Introduction

Groundwater is one of the most reliable natural resources for all the climatic zones across the global [1]. It plays an important role in people livelihood, agricultural production, industrial activities, socio-economic development, environment conservation etc. However, continuous increase in population, industrial expansion, more agricultural activities, excessive groundwater withdrawal and increased domestic use are resulting in the shortage of freshwater in many parts of the world [2,3]. Therefore, it is necessary to estimate precise amount of groundwater level for planning and management in water resource challenges, hydrologic modelling, climate change studies and sustainable development of the particular region [4].

There are several studies were noted by the numerous researchers using different modelling method for ground water level estimation at various scale [[5], [6], [7], [8], [9]]. These methods include physically models, experimental models, and numerical models such as finite difference, finite volume, finite element, and element-free methods. These models involve specific knowledge of the physical characteristics of the study area, complex boundary layers, more assumptions and large dataset which makes it more expensive, labour consuming, tedious etc [1,[10], [11], [12], [13]]. Nowadays, various machine learning techniques has been proved the capability to overcome the traditional techniques limitations and shown to be precise estimation of different parameters or events with multi time scale in the complex hydrology modelling studies [[14], [15], [16], [17], [18], [19], [20], [21], [22], [23], [24]].

Previously, many researchers documented the artificial neural network (ANN) as good substitute in both single and hybrid mode to mathematical model as its ability of quick process, less time consuming easy data handling and formulate the good hypothetical situations in complex hydrological processes [[25], [26], [27], [28], [29], [30], [31], [32]]. However, several researchers found that support vector machine (SVM) performs better than ANN for hydrological modelling [[33], [34], [35], [36], [37], [38], [39], [40]]. In context, Yoon et al. [39] used weighted error function method to enhance the capability of recursive prediction models based on SVM and ANN for groundwater depth forecasting in multiscale and found improved performance of SVM over than ANN. Similarly, Mirarabi et al. [40] compared the SVM and ANN, for groundwater levels prediction of confined and unconfined aquifers and found that the SVR model outperformed with R^2^ values 0.95, 0.61 and 0.79 at 1-, 2-, and 3-month ahead respectively than the ANN. Further, Liu et al. [41] proved the better ability of support vector machines with data assimilation (DA) method for estimation in groundwater behaviour in time scale data at Northeast United States. In recent years, various researches with deep learning have been done for the issue of groundwater level all over the world [[42], [43], [44], [45]]. Moreover, Solgi et al. [46] showed the efficacy of long short-term memory neural network (LSTM-NN) for groundwater level prediction and found the accuracy (R^2^) with values 99.89%, 99.00%, and 90.00% at 1-lag, 4-lags, and 26-lags ahead of groundwater level respectively, during the testing phase. Further, Wunsch et al. [47] used ANN, LSTM, convolutional neural networks (CNN), deep learning (DL) and non-linear autoregressive networks (NARX) for prediction of groundwater level and found that LSTM and CNN perform good with a larger dataset, where DL perform better with small dataset. Zhang and Zheng [48] predicted the depth to groundwater using coupled model of empirical mode decomposition (EMD) and LSTM and results showed that the prediction rate, maximum and minimum error is 100%, 5.00% and 0.07% respectively. The wavelet transform has also been studied for groundwater levels in various research studies [[49], [50], [51], [52]].

So far, many studies have been conducted in the field of using data preprocessing methods to increase modeling accuracy. In all the previous studies, the biggest flaws of the studies that have limited the use of their results are: (i) just several methods of data preprocessing methods have been investigated for a single study (ii) effect of data preprocessing methods have been investigated on just one of the artificial intelligence models (iii) not comparing the performance of data preprocessing methods in different types of artificial intelligence models at same study. However, in this research, the scientific and research gap of previous studies in this field will be covered, and many innovations have been proposed in this field, which have never been investigated in previous research. The main objectives of this study are: (i) to determine the impact of evaporation, precipitation, temperature, groundwater level parameters in modeling GWL values (ii) to develope a completely new hybrid model and how a POA-based bio-inspired optimization algorithm affects the performance of the artificial intelligence model (iii) to classify all available preprocessing methods in three different categories and to complete and accurate review and evaluation of the impact of preprocessing models in all types of artificial intelligence models (machine learning, deep learning and hybrid-ML models) at same study (iv) to identify the correct combination of artificial intelligence and signal decomposition techniques for predicting GWT values (v) to model and forecast the groundwater level of Hamedan-Bahar aquifer, which is one of the most important super-critical plains in Iran, with a variety of artificial intelligence models (machine learning, deep learning, and hybrid-ML models) (vi) to compare the effectiveness of using the best preprocessing methods in modeling the groundwater level with the state without using it. To achieve these objectives, GWL prediction outputs were analyzed using various statistical metrics and graphical indicators. Moreover, this research extends beyond theoretical exploration by emphasizing the practical implications of these findings. The novel insights derived from our analysis have direct relevance to stakeholders and decision-makers involved in groundwater management. By elucidating the strengths and limitations of various preprocessing methods, our work provides actionable information for optimizing predictive models in real-world applications. The potential for improving the accuracy of groundwater level predictions, as uncovered in this study, marks it as a pioneering effort with the capacity to influence not only current practices but also future research directions. In essence, the uniqueness of this study lies not only in its thorough methodology but also in its potential to bridge the gap between theoretical advancements and practical advancements in groundwater level prediction using artificial intelligence models.

## 2 Material and methods

### 2.1 Case study and data used

**Fig. 1:**
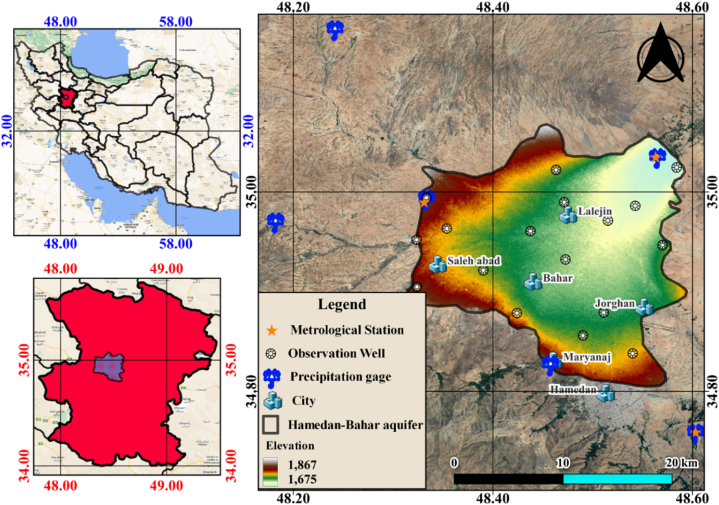
Location of the meteorological and precipitation stations and observation well in the case study area.

**Fig. 2:**
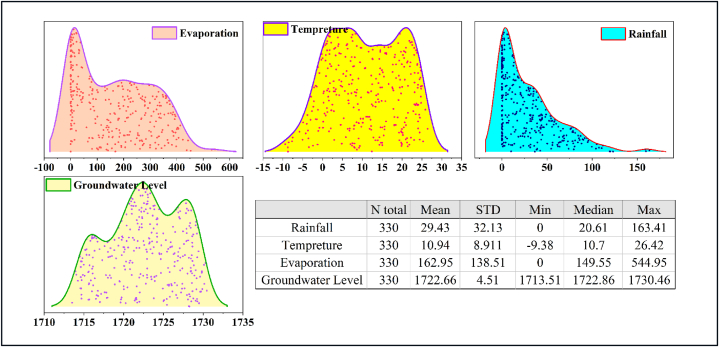
Statistical characteristics and ridgeline plot of the used data in the case study.

The Hamedan-Bahar Plain (HBP) serves as a principal aquifer and is amongst the largest plains of Iran, constituting a substantial part of the country's groundwater sources. Located northeast of the Alvand Mountains in the Hamedan province, the HBP, within the coordinates of 48° 33′E to 48° 58′ E and 34° 81′N to 35° 07′N, spans an area of 2463 km^2^ (Regional Water Company of Hamedan, 2023). Of this total area, the main unconfined alluvial aquifer, a vital component of the Hamedan-Bahar watershed, covers an expanse of 483 km^2^, with approximately 230 km^2^ dedicated to irrigation (Fig. 1). The HBP's elevation fluctuates between 1673 and 1871 m, with an average of 1739.25 m above the mean sea level. The groundwater data collected from 17 observational wells maintained by the Regional Water Company of Hamedan (RWCH) from September 1991 to February 2019 provide valuable insights into the groundwater levels and storage changes. The HBP's groundwater levels have demonstrated a significant decline at a 99% confidence level, with an average annual drop of 0.84 m from 1991 to 2018, as shown in Fig. 2. Also, Fig. 2 illustrates the locations of the monitoring wells within the HBP. Serving a crucial role in the agricultural sector of the Hamedan province, the HBP has been subjected to gradual subsidence in recent years, triggered by a marked drop in groundwater levels due to extensive extraction for agricultural, industrial, and drinking water purposes. The groundwater aquifers in the HBP remain unconfined, as depicted in Fig. 2, with descriptive statistics of the data inputs presented in the form of a ridgeline plot and table. For analysis purposes, the data sets are bifurcated into two classes: 70% for the training phase and the remaining 30% for the testing phase.

### 2.2 Pre-processing methods

There are numerous pre-processing methods which is widely used to remove noise of data, detrend of data, and change data scale that is helpful to achieve best model with high accuracy and less error. In this paper, input data for modelling is pre-processed by 126 methods which is classified in three level such as: 1- statistical, wavelet transforms, and decomposition methods which each of them will be described briefly in the next three sub-section.

#### 2.2.1 Statistical methods

This method is related to statistic science that is classified into six categories. Standardize method removes average of data and scale data to unit variance (Eq. (1)). Normalize method scales data to range between 0 and 1 value (Eq. (2)). Max absolute method scales data by its maximum value (Eq. (3)). Power transformer method uses Yeo-Johnson transform to make data like Gaussian (normal-like) format which 2 types of them is used that scale output to standardize and non-standardize (normalize) data [53] (Eq. (4)). Quantile method estimates cumulative distribution function (CDF) of data and map its value to a normal (Eq. (5)) or uniform distribution (Eq. (6)_. Robust scaler method removes the median and scales the data according to the quantile range which two value quantile range 5, 95 and 1, 99 are used in this research (Eq. (7)).

Standardize:

(1) $$X_{new}=\frac{X\;-\;\bar{X}}{\sigma }$$

Normalize:

(2) $$X_{new}=\frac{X-X_{\min}}{X_{\max}-\;X_{\min}}$$

Max absolute:

(3) $$X_{new}=\frac{X}{X_{\max}}$$

Power transformer:

(4) $$X_{new}=\left\{ \begin{array}{c} \frac{{\left(x_{i}+1\right)}^{\lambda }-1}{\lambda }\;if\;\lambda \neq 0,x\geq 0\;\\ \log\left(x_{i}+1\right)\;if\;\lambda =0,x\geq 0\\ \frac{{{-(-x}_{i}+1)}^{2-\lambda }-1}{2-\lambda }\;if\;\lambda \neq 2,x\;<0\;-\log\left(x_{i}+1\right)\;if\;\lambda =2,x\;<0 \end{array}\right.$$

Quantile transform:

(5) $$X_{new}=\sqrt{2}\sigma \times {\mathrm{erf}}^{-1}\left(2p-1\right)+\mu \;\text{Normal}\;\text{distribution}$$

(6) $$\left\{ \begin{array}{c} p=\frac{x-a}{b-a}\\ X_{new}=bp+a\left(1-p\right) \end{array}\right.\;\text{Uniform}\;\text{distribution}$$

Which ${\mathrm{erf}}^{-1}$ is the inverse error function, α is lower bond and b is upper bond of uniform distribution.

Robust scaler:

(7) $${\text{Remove}\;\text{median}\;\text{and}\;\text{outlier}\;\text{data},\text{then}\;X}_{new}=\frac{X\;-\;\bar{X}}{\sigma }$$

#### 2.2.2 Empirical mode decomposition (EMD)

In order to decompose the past total interference signal power, EMD uses the heuristic decomposition method to decompose it into intrinsic mode functions (IMF) [54]. Due to this method of decomposition, a frequency-ordered IMF component can be obtained. A high frequency oscillation is present in the initial IMFs. When the number of IMFs increases, the oscillation frequency decreases, resulting in more linear IMFs at the end. Using decomposed components, it is possible to recompute the total interference signal power (Eq. (8)).

(8) $$f\left(t\right)=\sum_{i=1}^{L-1}{IMF}_{i}\left(t\right)+res\left(t\right)$$

In this case, L represents the number of decomposed components, and the residual is denoted by res(t). Here, there is no predetermination of L in this case, and its value is determined solely by the nature of the signal. Developing and training a model to predict a signal with a high oscillatory frequency is more challenging than predicting a signal with a low oscillatory frequency. Due to this, rather than relying on a single forecasting model, the residual component and IMF components are developed separately. Here, increasing the IMF number results in a less oscillatory input signal to each model. This results in an improvement in the model's prediction accuracy as well as easier management of the training task. In the same manner as equation (8), by taking the individual predicted values of each IMF and residual, the anticipated value of total interference can also be reconstructed. Training and validation samples are obtained from the total interference signal (Eq. (9)). Splitting the training sample can be selected based on a predetermined percentage value (e.g., 70%).

(9) T=P+M

Where, in the total interference signal, T refers to the number of samples. Assume that P and M are the number of samples in the training data and the validation data, respectively.

#### 2.2.3 Wavelet transforms

The concept of a wavelet refers to the occurrence of a particular type of mathematical function (waveform) that oscillates and decays over time with a mean that is approximately zero. The scale components of a function are divided into different scale components, each of which can be assigned a specific frequency range. Wavelet analysis provides a means of displaying a time series in multiple resolutions. In order to match the outlines of time-series signals, the wavelet shape can be chosen or designed. There are three conditions that must be satisfied by a basis function in order for it to be considered a Wavelet [55].

i. Wavelet must have a mean value of zero (Eq. (10)).

(10) $$\int_{-\infty }^{\infty }\psi \left(t\right)dt=0$$

ii. It is essential that the wavelet be localized in both time and frequency space while maintaining a finite amount of energy (Eq. (11)).

(11) $$\int_{-\infty }^{\infty }{\left|\psi \left(t\right)\right|}^{2}d\left(t\right)<\infty$$

iii. Using inverse wavelet transforms, it is possible to reconstruct any signal x(n) of finite energy (admissibility condition) (Eq. (12)).

(12) $$C=\int_{-\infty }^{\infty }\frac{{\left|\psi \left(\omega \right)\right|}^{2}}{\left|\omega \right|}\;d\omega <\infty$$

In this case, ψ(t) represents the mother wavelet, and a sinusoid of appropriate frequency is given by ω.

In general terms, the Discrete Wavelet Transform (DWT) is commonly regarded as a discretized version of the Continuous Wavelet Transform. The utilization of DWT coefficients, which are typically associated with a single dyadic scale and time period [56,57], enables the reconstruction of any time-series through this form of representation. The intricacy of time series data gives rise to uncertainties in the high and low frequency components. Upon application of a DWT, the time-series data, commonly referred to as the signal, undergoes a decomposition process into discrete frequencies that operate at varying scales. Various classifications can be applied to categorize such transformations. Two functions that can be considered are the Scale Function and the Wavelet Function. The Scale Function is used to analyse signals across different frequency bands by breaking them down into broad and detailed components, similar to random uncertainty. On the other hand, the Wavelet Function is used to systematically dissect the coarse approximation, providing detailed insights similar to epistemic uncertainty. Moreover, High Pass and Low Pass filtering procedures address uncertainties that arise due to insufficient information regarding the parameters, which are akin to deep uncertainties. The aforementioned operations are linked to the scale and wavelet functions within the domain of frequency. The distinctive feature of the DWT lies in its ability to decompose a signal into non-sinusoidal constituents, thereby providing sufficient information for the analysis and synthesis of the original signal. This capability allows for the management of uncertainties that are intrinsically difficult to address in the context of time-series analysis. A detailed explanation of the mathematical background behind DWT can be found in various literature such as Percival [56] and Debnath and Shah [58].

#### 2.2.4 Variational mode decomposition (VMD)

According to VMD, the signal that will be analyzed is a straight-line superposition of several component modes. Band-limited intrinsic mode functions (BLIMF) are defined as frequency signals modulated by amplitude with as follows in Eq. (13) [59]:

(13) $$u_{k}=A_{k}\left(t\right)\mathrm{Cos}\;\varphi \left(t\right)$$

Where t represents the time; $u_{k}$ represents the kth BLIMF; An instantaneous amplitude is given by $A_{k}\left(t\right)$; and the phase of the signal is indicated by φ(t). To construct the variational modes, the Hilbert transform is used to calculate the one-sided spectrum of each BLIMF component, which is then used to estimate the central frequency (Eq. (14)) [59]:

(14) $$\left\{ \begin{array}{c} \begin{array}{l} \min\\ \left\{u_{k},\omega_{k}\right\} \end{array}\{\sum_{k=1}^{K}\|\partial_{t}\left[\left(\sigma \left(t\right)+\frac{j}{\pi t}\right)⨂\;u_{k}\left(t\right)\right]e^{-jw_{k}t\|\begin{array}{c} 2\\ 2 \end{array}}\\ s.t.\sum_{k=1}^{K}u_{k}\left(t\right)=f\left(t\right) \end{array}\right.$$

where k = 1,2, …, K; A unit pulse function is defined as σ(t); $\omega_{k}$ is the frequency of the center; An input signal is given by f(t); An indication of the convolution operation is provided by ⨂; Partial derivative is represented by $\partial_{t}$; and a number with imaginary value j is used.

In order to obtain the extended Lagrange expression, penalty factors are introduced and the Lagrange multiplier operator is introduced to turn the constrained variational problem into an unconstrained variational problem (Eq. (15)) [59].

(15) $$\left\{ \begin{array}{c} L\left(\left\{u_{k}\right\},\left\{\omega_{k}\right\},\lambda \right)=\alpha \sum_{k}\|\partial_{t}\left[\left(\delta \left(t\right)+\frac{j}{\pi t}\right)*\;u_{k}\left(t\right)\right]e^{-jw_{k}t\|\begin{array}{c} 2\\ 2 \end{array}}\\ +\|f\left(t\right)-\sum_{k}u_{k}\left(t\right)\|\begin{array}{c} 2\\ 2 \end{array}+\left\langle \lambda \left(t\right),f\left(t\right)-\sum_{k}u_{k}\left(t\right)\;\right\rangle \end{array}\right.$$

Where a bandwidth parameter is given by α.

A continuous updating of the center frequency and bandwidth is performed during the solution phase until the iteration-stopping condition is met for each component (Eq. (16)) [59].

(16) $$\sum_{k=1}^{K}\left(\left\|{\hat{u}\;}_{k}^{n+1}-{\hat{u}\;}_{k}^{n}\right\|\|\begin{array}{c} 2\\ 2 \end{array}\;/\;{\hat{u}\;}_{k}^{n}\|\begin{array}{c} 2\\ 2 \end{array}\;\right)<\emptyset$$

Where $\hat{u}$ represents the expression after the kth update to BLIMF; and ∅ represents the discriminative accuracy, which is generally assumed to be 10-6. A Fourier transform in the inverse direction is applied in order to convert the signal frequency domain characteristics to the time domain at the end of the iteration.

### 2.3 Artificial intelligence models

Models of artificial intelligence work by recognizing patterns in training data and making predictions or decisions based on those patterns. AI models perform better in their data analysis and forecasting when they receive more data points. The application of AI modeling involves the design, training, and deployment of machine learning algorithms that are intended to emulate the logic of logical decision making based on data sets that are available. Models based on artificial intelligence provide a basis for advanced intelligence techniques, such as real-time analytics, predictive analyses, and augmented analyses.

#### 2.3.1 Support vector regression (SVR)

The Support Vector Regression (SVR) algorithm performs regression analysis and classification tasks using a subset of the support vector machine (SVM) technique [60]. It works well with data that can be partitioned linearly, but it uses a nonlinear mapping technique to transform data that cannot be partitioned linearly into a higher-dimensional feature space. Using a linear algorithm in the higher-dimensional space becomes possible after this transformation is applied. To achieve global optimization in supervised learning, SVR seeks to reduce structural risk and locate the best possible classification surface. It guarantees that the calculated risk is consistent with the probabilities. To generate a classification hyperplane, SVR uses statistical risk reduction to maximize margins across classes. Similar to the goal of ordinary linear regression, which is to reduce the residual sum of squares, SVR in regression analysis seeks a hyperplane that minimizes the distance to all data points. In SVR, kernel functions are used to improve the search for the best hyperplane by projecting a non-linear regression issue into a higher-dimensional space.

(17) $$\max\;\left[-\frac{1}{2}\sum_{i=1}^{k}\sum_{i=j}^{k}\left(a_{i}-a_{i}^{*}\right)\left(a_{j}-a_{j}^{*}\right)K\left(X_{i},X_{j}\right)-\;\sum_{i=1}^{k}\left(a_{i}+a_{i}^{*}\right)\varepsilon +\sum_{i=1}^{k}\left(a_{i}-a_{i}^{*}\right)Y_{i}\right]s.t\left\{ \begin{array}{c} \sum_{i=1}^{k}\left(a_{i}-a_{i}^{*}\right)=0\\ \begin{array}{c} 0\leq a_{i},a_{i}^{*}\leq \frac{C}{l}\\ i=1,2,\ldots ,l \end{array} \end{array}\right.$$

Where, the sample data is $X_{i}$; Sample size is given by l; a penalty coefficient is expressed as C; A size of ε exceeds the size of the penalty sample for the error; and kernel function is referred to as $K\left(X_{i},X_{j}\right)$.

In the case of set a = [$a_{1},a_{1}^{*},\ldots ,a_{1},a_{j}^{*}]$, it is the optimal solution, and this equation for the SVR regression can be expressed as follows:

(18) $$f\left(x\right)=\sum_{i=1}^{k}\left(a_{i}-a_{i}^{*}\right)K\left(X_{i},X_{j}\right)+b^{*}$$

The parameters of the part $\left(a_{i}-a_{i}^{*}\right)$ must not be equal to zero. the corresponding samples $X_{i}$ will be the support vectors in this problem.

#### 2.3.2 Artificial neural network (ANN)

The Artificial Neural Network (ANN) is an effective non-linear modeling technique based on the workings of a human brain. Analysing input datasets and output values in order to identify and learn interconnected patterns can be accomplished using ANN. A simple neural network can be defined as a collection of simple neurons or nodes that carry out simple numerical operations interconnected in a specific manner. ANN normally consist of three layers and a number of nodes. It consists of three layers: the input layer, comprising of different input parameters, the hidden layer, consisting of a number of hidden neurons, and the single output layer, which represents a target value [61]. A multilayer perceptron (MLP) is the most commonly used artificial neural network (ANN) for modeling hydrological processes due to its potential for non-linear pattern recognition and memory association [[62], [63], [64]]. Neurons in the MLP are arranged in layers and are simply associated with neurons in adjacent layers.

An ANN model aims to generalize a relationship in the following order (Eq. (19)):

(19) $$Y^{m}=f\left(X^{n}\right)$$

Assuming that $X^{n}$ is a vector of n-dimension consisting of variables $x_{1},x_{2},\ldots ,x_{i},\ldots ,x_{n}$; whereas $Y^{m}$ output vector of m-dimension comprising of resultant variables of interest $y_{1},y_{2},\ldots ,y_{i},\ldots ,y_{n}$.

The neural network is composed of layers *i*, *j*, and *k* with inter-connection weights w_ij_ and w_jk_ between the layers of neurons. Each neuron in a layer receives and analyses weighted input from the preceding layer and then transmits its output via links to nodes in the subsequent layer. The weight coefficient *w*_*ij*_ describes the link between the *i*th and *j*th neurons, while the threshold coefficient *b* describes the *i*th neuron. The weight coefficient indicates the significance of a particular connection in the network. The *i*th neuron's output value is calculated as follows (Eqs. (20), (21))) [65,66]:

(20) $$x_{i}=f\left(\in_{i}\right)$$

(21) $$\in_{i}=b_{i}+\sum_{j^{\epsilon }\Gamma_{i}^{-1}}w_{ij}x_{j}$$

Where, $f\left(\in_{i}\right)$ = activation function. With formally added neuron j, where x_j_ = 1, Sigmoid shape activation functions are typically defined as (Eq. (22)):

(22) $$f\left(\in_{i}\right)=\frac{1}{1+e^{-\in }}$$

The weight updates are calculated using the following formula (Eq. 23):

(23) $${\Delta w}_{ij}\left(t\right)=-\eta \frac{\partial E}{{\partial w}_{ij}}+\mu {\Delta w}_{ij}\left(t-1\right)$$

where, *η* = learning rates and μ = momentum rates, *E* = error or objective function, and ${\Delta w}_{ij}\left(t\right)$ = weight increments between nodes *i* and *j* for iterations *t* and ${\Delta w}_{ij}\left(t-1\right)$ are the weight increments between nodes *i* and *j* for iterations *t-1*.

#### 2.3.3 Long short-term memory (LSTM)

**Fig. 3:**
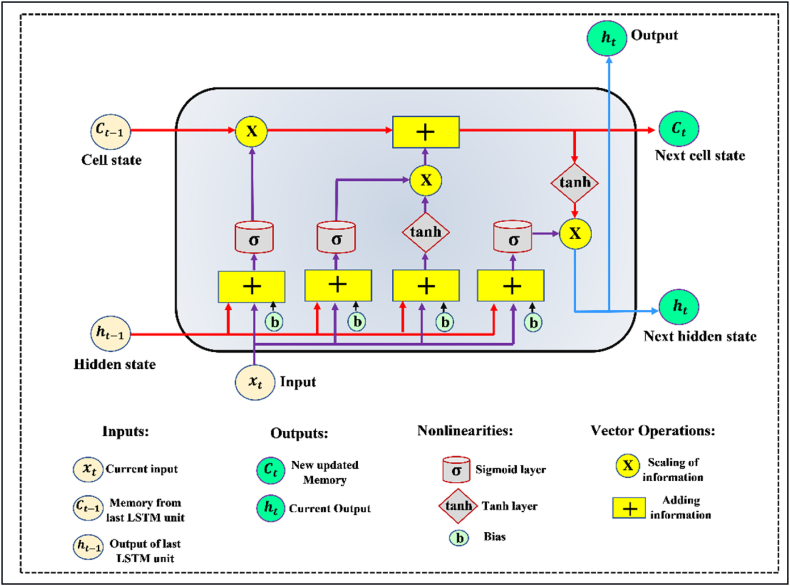
General structure of LSTM model.

Hochreiter and Schmidhuber [67] introduced what is referred to as a long-term short-term memory (LSTM), as an improvement on a Recurrent Neural Network (RNN), by adding in addition to the basic interactions occurring in each module (or cell) of the RNN, to address the aforementioned deficiencies of the RNN. An LSTM is a type of RNN that has the capability of learning long-term dependencies and remembering information for extended periods of time, by default. Models based on LSTMs are structured in a chain structure. Nevertheless, the repeating module is structured differently. It consists of four interacting layers rather than a single neural network, as is the case with a standard RNN. A detailed illustration of the structure of the LSTM neural network can be found in Fig. 3.

LSTM networks are typically composed of memory blocks known as cells. The cell state as well as the hidden state are being transferred to the next cell. Data flow is mainly defined by the cell state, which allows data to pass essentially unchanged from one point to another. It is possible, however, to experience some linear transformations. A sigmoid gate allows for the addition or removal of data from the cell state. In the case of gates, they are similar to layers or multiple matrices containing different individual weights. By utilizing gates to control the learning process, LSTM avoid the long-term dependency problem.

A LSTM network is constructed by first identifying the information that is not required for the network to be constructed and omitting that information from the cell in the first step of the construction process. The sigmoid function is used in this process to determine which data should be included and excluded, as it takes the output from the last LSTM unit (h_t−1_) at time t−1 and the current input (X_t_) at time t (Eq. (24)). As a consequence, the sigmoid function also determines which part of the output from the old output should be eliminated as well. The gate is known as the forget gate (or ft); Where ft is a vector containing a range of values from 0 to 1, corresponding to each number in the cell state, C_t−1_.

(24) $$f_{t}=\sigma \left(W_{f}\left[h_{t-1},X_{t}\right]+b_{f}\right)$$

Assuming that σ is a sigmoid function, and the forget gate has two matrices, $W_{f}$ and $b_{f}$, representing its weights and bias, respectively. Afterward, information from the new input (X_t_) is decided and accumulated in the cell state, as well as the cell state is updated. There are two parts to this step, the first being the sigmoid layer, and the second being the tanh layer. To begin with, the sigmoid layer determines whether the new information should be updated or ignored (0 or 1) (Eq. (25)), Also, the tan(*h*) function determines the importance level of the values that pass by, based on their weight (−1 to 1) (Eq. (26)). To update the state of the cell, the two values are multiplied. As a result, C_t_ is formed by adding the new memory to the old memory C_t−1_ (Eq. (27)).

(25) $$i_{t}=\sigma \left(W_{i}\left[h_{t-1},X_{t}\right]+b_{i}\right)$$

(26) $$N_{t}=\tan\;h\left(W_{n}\left[h_{t-1},X_{t}\right]+b_{n}\right)$$

(27) $$C_{t}=C_{t-1}f_{t}+N_{t}i_{t}$$

Here, $C_{t-1}$ and $C_{t}$ are the cell states at time t−1 and t; the weight matrix and bias of the state of the cell are represented by W and b. As a final step, the output values (h_t_) are filtered version based on the output cell state (O_t_). The first step is to determine which parts of the cell state will be output by a sigmoid layer (Eq. (28)). Next, the output of the sigmoid gate (O_t_) is multiplied by the new values created by the tanh layer from the cell state (C_t_), with a value ranging between −1 and 1 (Eq. (29)).

(28) $$O_{t}=\sigma (W_{0}\left[h_{t-1},X_{t}\right]+b_{0}$$

(29) $$h_{t}=O_{t}\;\tanh\left(C_{t}\right)$$

Where, $W_{0}$ and $b_{0}$ represent the weight matrix and bias of the output gate, respectively.

### 2.4 Pelican optimization algorithm (POA)

Trojovský and Dehghani [68] proposed a novel metaheuristic optimization algorithm based on swarm intelligence as a means of solving the optimization problem; this is the pelican optimization algorithm (POA), which has been described as a metaheuristic algorithm based on swarm intelligence. Pelicans often hunt in groups for prey such as fish, which inspired the development of the algorithm. There is a great deal of wisdom in pelicans' hunting behaviour. During hunting, pelicans find prey in advance and approach it quickly, completing the behaviour in a swotting posture when the distance between them is between 10 and 20 m [69]. Here are the steps that can be taken to represent the detailed framework of the POA:

***Population initialization:*** There is a certain range of search space within which pelicans generally seek out prey, so the initial position of each pelican in the population is determined at random, and it can be expressed as follows in Eq. (30):

(30) $$P_{i}=S_{\min}+rand\;.\;\left(S_{\max}-\;S_{\min}\right)\;i=1,\ldots ,I$$

Here, $P_{i}$ represents the pelican's initial position. There are a maximum number of pelicans indicated by I. Minimum and a maximum boundary are defined respectively by $S_{\min}$ and $S_{\max}$. A random number in the range (0, 1) is represented by rand.

***Exploration phase:*** During this stage, pelicans are primarily focused on locating and determining the location of their prey and preparing for an attack. As a result, Eq. (27) is used to update the position of each pelican:

(31) $$P_{i}^{1}=\left\{ \begin{array}{c} P_{i}+rand\;.\;\left(P_{p}-U.P_{i}\right),f_{p}<\;f_{i}\\ P_{i}+rand\;.\;\left(P_{i}-\;P_{p}\right),else \end{array}\right.$$

Here, $P_{i}^{1}$ represents the updated position of the pelican, while $P_{p}$ represents the position of the prey during the exploration phase. It is determined that $f_{p}$ represents the objective function of the prey and $f_{i}$ represents the objective function of the pelican. Additionally, U may be either a 1 or a 2 based on randomness.

***Exploitation phase:*** As soon as the pelican is in an advantageous position and begins to attack, the fish is propelled into the throat pouch of the pelican by the pelican's flight across the water. As a mathematical expression, this strategy is as follows in Eq. (32):

(32) $$P_{i}^{2}=P_{i}+z\;.\;\left(1-\frac{t}{T}\right).\;\left(2.rand-1\right)\;.\;P_{i}$$

During the exploitation phase, $P_{i}^{2}$ represents the pelican's updated position. The value of z is a random number between 0 and 2. In this case, T represents the maximum number of iterations, and the current iteration is represented by t.

***Limitations of POA algorithm:*** It is important to note that the POA algorithm does have certain limitations, just as any other optimization algorithm does. It is, therefore, important to consider the POA's limitations when deciding which algorithm to use for a given optimization problem, despite its potential for certain optimization problems. It is generally possible to express the limitations of the POA algorithm as follows.

1 Lack of scalability: Increasing the search space and the number of pelicans can make the POA more computationally expensive. Due to this limitation, the algorithm may not be as effective for large-scale optimization problems.
2 Computationally intensive: Due to its population-based approach to solving optimization problems, the POA is computationally intensive. When computational resources are limited in real-world applications, this may limit its practicality.
3 Limited to continuous search space: In terms of search spaces, the POA is designed for continuous searches. In the case of discrete search spaces, which are commonly encountered in optimization tasks, it may not be suitable.

### 2.5 Hybrid model (POA-ANN)

The procedure for implementing the Hybrid Particle Optimization Algorithm - Artificial Neural Network (POA-ANN) model involves incorporating the ANN into a single framework integrated with the POA. The standalone ANN model, however, encounters challenges like falling into local minima and sluggish learning rates. To that end, using optimization algorithms such as the POA in conjunction with ANN can markedly improve the ANN's performance concerning these setbacks. The blended POA-ANN approach accomplishes its objective in two steps.

• Utilizing the POA technique to enhance the ANN's structure and its variables.
• Procuring the optimal response through ANN.

**Fig. 4:**
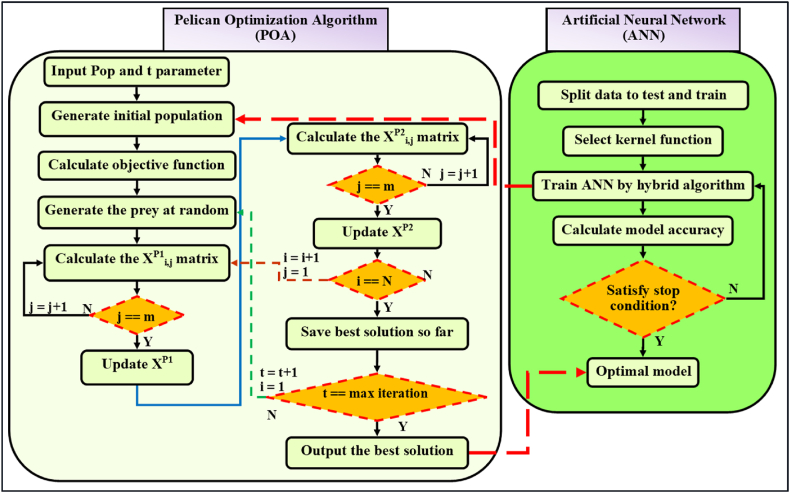
Flowchart of the POA-ANN model.

**Fig. 5:**
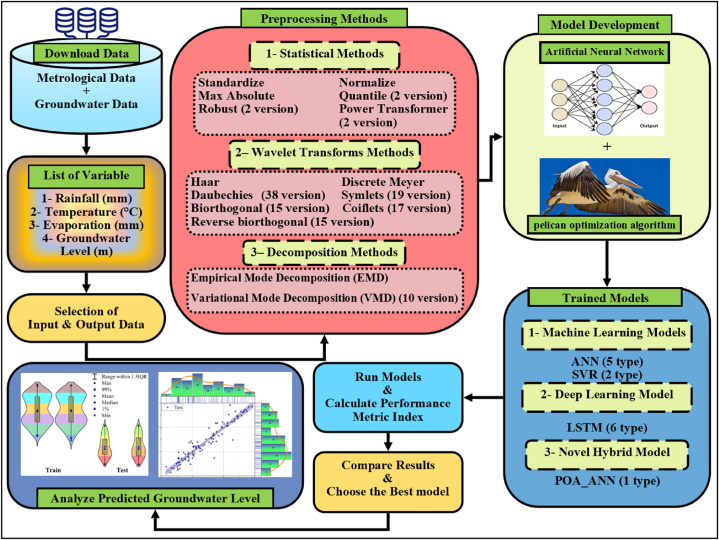
Schematic diagram of the proposed methodology.

**Fig. 6:**
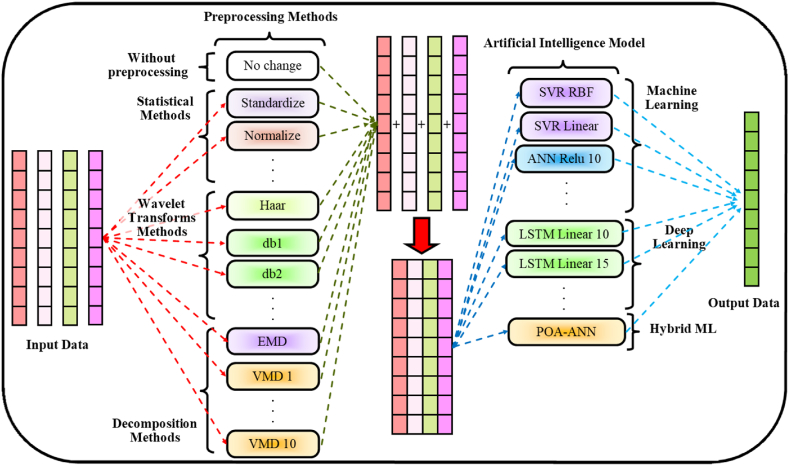
Schematic diagram of the model structure and how to process data.

In the course of this research, the POA method was preferred to maximize the optimal count of hidden neurons, weights, and bias values pertinent to the ANN models. The variables of the POA, including the likelihood of crossover, selection methodology, mutation rate, size of the population, and the count of generations, were evaluated following a hit and trial procedure; specifics concerning POA parameters are detailed in Table 3. The flow chart illustrating the proposed hybrid POA-ANN strategy is displayed in Fig. 4. Fig. 5 presents a schematic and overall representation of the proposed method, along with the inputs and phases of modeling employed in this research. As shown in Fig. 6, more detailes on model structure and data processing of this study were depicted.

### 2.6 Performance metric index

The predictive models are evaluated using by just one performance criteria, because of lack of space and limitation to present results. In this paper, Akaike information criterion (AIC) was used as a reference indicator to compare the performance of different pre-processing methods in different models (Eq. (33)). Many research studies have used the Akaike information criterion (AIC) index as a valid and reliable method of predicting and estimating groundwater levels [[70], [71], [72], [73], [74]].

Akaike information criterion (AIC):

(33) $$\text{AIC}=N\times \ln\left(\frac{\sum {\left(O-\;P\right)}^{2}}{N}\right)+2k$$

where, O and P are the observed and predicted values, N is the number of observations, and $\bar{O}$ and $\bar{P}$ are the average of the observed and predicted value, respectively. k is number of parameters.

## 3 Results and discussion

### 3.1 Results

In this study, various meteorological (precipitation, temperature, and evaporation) and GWL data were used to estimate one-month lead time GWL values in Hamedan-Bahar Plain, Iran. ANN, LSTM and POA-ANN models were combined with various Pre-processing techniques, EMD, Wavelet and VMD methods to estimate GWL values. Established model scenarios are shown in Table 1. Accordingly, the performances of the stand-alone ML and DL models were compared with the DL combined with bio-inspired optimization. In addition, it has been analyzed how the GWL prediction performance of the model changes when various pre-processing and data decomposition techniques are applied to the stand-alone models. In addition, it was investigated that the predictive power was increased by integrating the mother wavelet and the VMD version into the ML and DL techniques.

**Table 1:** Table of models and scenarios for input (P_t_, T_t_, E_t_, GWL_t_) and output (GWL_t+1_).

Pre-processing		Model													
		Machine Learning (ML)							Deep Learning (DL)						Hybrid ML
		SVR		ANN					LSTM						POA-ANN
		RBF	Linear	Relu_10	Relu_15	Relu_20	Relu_25	Relu_30	Linear_10	Linear_15	Linear_20	Relu_10	Relu_15	Relu_20	Relu_10
No Change		S_1	S_128	A_1	A_128	A_255	A_382	A_509	L_1	L_128	L_255	L_382	L_509	L_636	P_1
Standard		S_2	S_129	A_2	A_129	A_256	A_383	A_510	L_2	L_129	L_256	L_383	L_510	L_637	P_2
Normalize		S_3	S_130	A_3	A_130	A_257	A_384	A_511	L_3	L_130	L_257	L_384	L_511	L_638	P_3
Max Absolute		S_4	S_131	A_4	A_131	A_258	A_385	A_512	L_4	L_131	L_258	L_385	L_512	L_639	P_4
Power Transformer (Yeo-Johnson)		S_5	S_132	A_5	A_132	A_259	A_386	A_513	L_5	L_132	L_259	L_386	L_513	L_640	P_5
Power Transformer (Yeo-Johnson, non-standardize)		S_6	S_133	A_6	A_133	A_260	A_387	A_514	L_6	L_133	L_260	L_387	L_514	L_641	P_6
Quantile (normal)		S_7	S_134	A_7	A_134	A_261	A_388	A_515	L_7	L_134	L_261	L_388	L_515	L_642	P_7
Quantile (uniform)		S_8	S_135	A_8	A_135	A_262	A_389	A_516	L_8	L_135	L_262	L_389	L_516	L_643	P_8
Robust (5, 95)		S_9	S_136	A_9	A_136	A_263	A_390	A_517	L_9	L_136	L_263	L_390	L_517	L_644	P_9
Robust (1, 99)		S_10	S_137	A_10	A_137	A_264	A_391	A_518	L_10	L_137	L_264	L_391	L_518	L_645	P_10
EMD		S_11	S_138	A_11	A_138	A_265	A_392	A_519	L_11	L_138	L_265	L_392	L_519	L_646	P_11
Wavelet Transform	haar	S_12	S_139	A_12	A_139	A_266	A_393	A_520	L_12	L_139	L_266	L_393	L_520	L_647	P_12
	db1	S_13	S_140	A_13	A_140	A_267	A_394	A_521	L_13	L_140	L_267	L_394	L_521	L_648	P_13
	–	–	–	–	–	–	–	–	–	–	–	–	–	–	–
	sym2	S_51	S_178	A_51	A_178	A_305	A_432	A_559	L_51	L_178	L_305	L_432	L_559	L_686	P_51
	–	–	–	–	–	–	–	–	–	–	–	–	–	–	–
	coif1	S_70	S_197	A_70	A_197	A_324	A_451	A_578	L_70	L_197	L_324	L_451	L_578	L_705	P_70
	–	–	–	–	–	–	–	–	–	–	–	–	–	–	–
	bior1.1	S_87	S_214	A_87	A_214	A_341	A_468	A_595	L_87	L_214	L_341	L_468	L_595	L_722	P_87
	–	–	–	–	–	–	–	–	–	–	–	–	–	–	–
	rbio1.1	S_102	S_229	A_102	A_229	A_356	A_483	A_610	L_102	L_229	L_356	L_483	L_610	L_737	P_102
	–	–	–	–	–	–	–	–	–	–	–	–	–	–	–
	dmey	S_117	S_244	A_117	A_244	A_371	A_498	A_625	L_117	L_244	L_371	L_498	L_625	L_752	P_117
VMD	1	S_118	S_245	A_118	A_245	A_372	A_499	A_626	L_118	L_245	L_372	L_499	L_626	L_753	P_118
	–	–	–	–	–	–	–	–	–	–	–	–	–	–	–
	10	S_127	S_254	A_127	A_254	A_381	A_508	A_635	L_127	L_254	L_381	L_508	L_635	L_762	P_127
		**S: SVR**		**A: ANN**		**L: LSTM**		**P: POA-ANN**							

Table 2 shows the mother wavelet types and vanishing moments used for the sub-signal separation of the inputs. Accordingly, it was evaluated which mother wavelet produced more effective outputs in semi-arid shadows for GWL estimation.

**Table 2:** Wavelet properties.

Full name of wavelet transforms	Abbreviation	List of used them
Haar	haar	haar
Daubechies	db	db1, db2, db3, db4, …, db38
Symlets	*sym*	sym2, sym3, sym4, …, sym20
Coiflets	coif	coif1, coif2, coif3, …, coif17
Biorthogonal	bior	bior1.1, bior1.3, bior1.5, bior2.2, bior2.4, bior2.6, bior2.8, bior3.1, bior3.3, bior3.5, bior3.7, bior3.9, bior4.4, bior5.5, bior6.8
Reverse biorthogonal	rbior	rbio1.1, rbio1.3, rbio1.5, rbio2.2, rbio2.4, rbio2.6, rbio2.8, rbio3.1, rbio3.3, rbio3.5, rbio3.7, rbio3.9, rbio4.4, rbio5.5, rbio6.8
Discrete Meyer	dmey	dmey

**Table 3:** Parameters used for training models.

Models	Type of parameters	Value
SVR	Data Division	Split train and test (70, 30)
	Kernel Type	RBF, Linear
ANN	Network Type	feed-forward back propagation
	Data Division	Split train and test (70, 30)
	Number of Hidden layer (Neurons)	10, 15, 20, 25, 30
	Epoch number	1000
	Learning rate	0.001
	Activation Function	Relu
	Training function	Adam
LSTM	Data Division	Split train and test (70, 30)
	Number of Hidden layer (Neurons)	10, 15, 20
	Epoch number	400
	Learning rate	0.001
	Activation Function	Linear, Relu
	Training function	Adam
POA-ANN	Max Iteration	500
	Population	100
	Number of Hidden layer (Neurons)	10
	Activation Function	Relu

Table 3 presents the parameters for the ML, DL and POA algorithms. The selected parameters significantly affect the performance of the models and are used to optimize the model. For this reason, various hidden layer neuron numbers, iteration numbers and activation functions were tried during the training (Table 3).

**Fig. 7:**
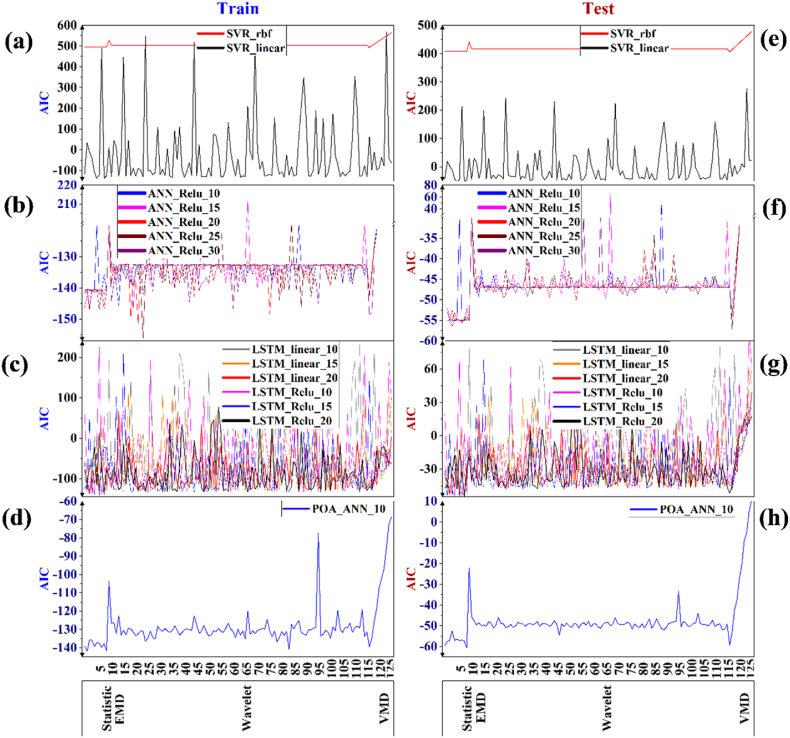
AIC performance value of the applied models for each group of AI models based on pre-processing methods - which are numbered from 1 to 127 (based on Table 1) - for both training (a, b, c, and d) and testing (e, f, g, and h) phases. (a & e) SVR, (b & f) ANN, (c & g) LSTM, and (d & h) hybrid ANN.

Fig. 7 shows the variation of the AIC values of the applied models of each model type (including: SVR, ANN, LSTM, Hybrid-ANN) for the training (a, b, c, and d) and testing (e, f, g, and h) phases. The model with the lowest AIC value was rated as the best. Accordingly, while the most accurate estimations are obtained with the ANN and POA-ANN models, it is noteworthy that the weakest estimates are in the SVM models. In addition, Linear SVM among SVMs, Relu_20 and Relu_20 models among ANNs, and Relu_20 and Linear 20 models among LSTMs were the best. In addition, it is seen that the wavelet transform approach is superior to other pre-processing techniques with its lower AIC values. Furthermore, in the VMD (Number 118–128) and EMD (Number 11) section on X-axis, there is a large fluctuation in all model types which shows that their performance was weaker than other pre-processing techniques.

The average value of the total changes of the AIC index for all models in all three groups shows a sharp decrease (Table 4). The value of this decrease is −25%, −29% and −57% for the statistical, wavelet, and decomposition classes, respectively, among which, the statistical method class has the smallest decrease in changes and the decomposition class has the most quantitative changes. This reduction procedure has also been established for all other AI models except the SVR model, in which the wavelet pre-processing method has the least reduction of changes and the decomposition method has the most value changes. Also, this procedure is also true for the highest influence, so that the highest influence belongs to the statistical, wavelet and decomposition method, respectively, with the values of 141%, 121% and 119%.

**Table 4:** Percent change value of summation of AIC indicators for each class of AI models (%).

Type	Class type	AI models class				Total
		ML	DL		Hybrid-ML	
		SVR	ANN	LSTM	POA-ANN	
**mean**	Statistical	−0.441	−0.014	−0.428	−0.015	−0.253
	Wavelet	−0.413	−0.092	−0.461	−0.098	−0.296
	DM	−0.668	−0.346	−0.779	−0.352	−0.578
**max**	Statistical	0.215	0.040	1.415	0.020	1.415
	Wavelet	0.142	0.052	1.217	−0.055	1.217
	DM	0.004	0.035	1.199	0.002	1.199
**Best method based on max value**	Statistical	Power Transformer (yeo-johnson, standardize)	Standard Scaler	Quantile Transformer (normal)	Robust Scaler (1, 99)	Quantile Transformer (normal)
	Wavelet	db25_1	db13_1	db9_1	db14_1	db9_1
	Decomposition method	VMD_1	VMD_1	VMD_2	VMD_1	VMD_2

Based on average value for summation of AIC value, all the pre-processing methods in SVR and LSTM models have the least impact, but in ANN and hybrid models, they have the most impact, which shows the extreme complexity of the LSTM model and the linear and non-linear mapping of the SVR model are reasons that cause the accuracy of the model to be greatly reduced by changing the orginal data. The average ratio of superiority of different pre-processing classes in all AI models is that in statistical, wavelet and decomposition pre-processing class in the group of neural network models (ANN and POA-ANN) are, respectively, approximately 30, 4.5 and 2 times better than the other group (SVR and LSTM).

Unlike the past, based on max value for summation of AIC value, almost all pre-processing methods in SVR and LSTM models have the highest influence, but in ANN and hybrid models, they have the lowest impact, which means that for this type of models, choose the best pre-processing methods by trial and error are the best way. The average ratio of superiority of different pre-processing classes in all AI models is that in statistical, wavelet and decomposition pre-processing class in the group of none neural network models (SVR and LSTM) especially LSTM model are, respectively, approximately 30, 600 and 35 times better than the neural network group (ANN and POA-ANN). This result for LSTM model show that select the best pre-processing methods for this AI model has a vital importance on predicted result.

Results of the best method for each pre-processing class indicate that: 1- the best methods in statistical class are very diverse from robust scale to quantile transformer (normal), so that standardization methods have a better chance of being superior. 2- the best methods in wavelet transform class are unpredictable and different types of it should be subject to trial and error in order to get the best one, but it can be indicated that the types related to the Daubechies group have a much better performance than other groups. 3- the best methods in decomposition class are belongs to the VMD type and also, from the results of the table, it can be interpreted that the lower levels of decomposition have performed much better in this method.

Table 5 shows the model performance ranking according to the AIC indicators. Accordingly, the model with a lower AIC value was interpreted as being superior. The best models for estimating GWL values according to AIC values were obtained with the Relu activation function of the ANN model, 25 and 30 hidden layer neurons. In addition, Normalized data pre-processing, db mother wavelet and VMD decomposition techniques were applied to these models, respectively. The weakest GWL predictive results were obtained by combining SVM and VMD techniques with RBF activation function.

**Table 5:** Models’ ranks based on summation AIC performance metric index for train and test in each group.

Rank	Model													
	SVR		ANN					LSTM						POA-ANN
	RBF	Linear	Relu_10	Relu_15	Relu_20	Relu_25	Relu_30	Linear_10	Linear_15	Linear_20	Relu_10	Relu_15	Relu_20	Relu_10
1	S_118	S_133	A_118	A_245	A_372	A_406*	A_511**	L_7	L_132	L_257	L_386	L_515	L_644	P_10
2	S_1	S_136	A_1	A_128	A_257	A_499	A_626***	L_2	L_150	L_263	L_389	L_512	L_749	P_2
3	S_2	S_164	A_8	A_129	A_263	A_388	A_515	L_27	L_134	L_262	L_384	L_510	L_703	P_118
4	S_3	S_184	A_4	A_132	A_277	A_391	A_517	L_94	L_155	L_304	L_414	L_529	L_685	P_1
5	S_4	S_169	A_10	A_136	A_274	A_383	A_509	L_17	L_208	L_309	L_478	L_592	L_674	P_8
…	…	…	…	…	…	…	…	…	…	…	…	…	…	…
123	**S_123**	S_135	A_125	A_251	A_377	A_504	A_632	L_112	L_243	L_350	L_409	L_589	L_708	P_97
124	**S_124**	S_198	A_6	A_252	A_378	A_505	A_633	L_11	L_164	L_303	L_497	L_522	L_671	P_124
125	**S_125**^**###**^	S_173	A_126	A_253	A_379	A_506	A_634	L_40	L_160	L_381	L_473	L_626	L_689	P_125
126	**S_126**^**##**^	S_153	A_127	A_254	A_380	A_507	A_635	L_120	L_146	L_269	L_507	L_596	L_713	P_126
127	**S_127**^**#**^	S_252	A_89	A_195	A_381	A_508	A_565	L_114	L_253	L_342	L_388	L_525	L_691	P_127
**S: SVR** **A: ANN**	**L: LSTM** **P: POA-ANN**		* Best 1	** Best 2	*** Best 3	^**###**^**Worst 3**	^**##**^**Worst 2**	^**#**^**Worst 1**	No change type	Statistical type	EMD type	Wavelet type	VMD type	

Table 6 compares the observed and predicted values of the first-order model to estimate the GWL at nine extreme values. The table contains a side-by-side comparison of the observed and predicted values of the model and helps assess any discrepancy between the two. In addition, the power of the model and its success in accurately predicting the GWL under different conditions are analyzed. Accordingly, the POA-ANN hybrid technique represented the observation data best, with the lowest error values in the GWL estimation.

**Table 6:** Compare observation and 1st rank models GWL prediction value at nine extreme values on verification (test) period.

Month	Obs	Model													
		SVR		ANN					LSTM						POA-ANN
		RBF	Linear	Relu_10	Relu_15	Relu_20	Relu_25	Relu_30	Linear_10	Linear_15	Linear_20	Relu_10	Relu_15	Relu_20	Relu_10
		S_118	S_133	A_118	A_245	A_372	A_406	A_511	L_7	L_132	L_257	L_386	L_515	L_644	P_10
6	1719.61	1724.57	1721.79	1721.88	1721.88	1721.88	1721.75	1721.79	1721.99	1721.85	1721.92	1721.95	1721.86	1721.97	1721.65
10	1719.89	1723.95	1717.46	1717.53	1717.53	1717.52	1717.44	1717.51	1717.54	1717.46	1717.55	1717.60	1717.51	1717.56	1717.61
30	1717.61	1724.54	1718.72	1718.81	1718.81	1718.81	1718.92	1718.91	1718.84	1718.74	1718.86	1718.87	1718.79	1718.84	1718.97
34	1718.11	1724.03	1715.20	1715.27	1715.27	1715.26	1715.22	1715.23	1715.34	1715.24	1715.25	1715.34	1715.25	1715.35	1715.35
40	1718.26	1725.30	1719.54	1719.63	1719.63	1719.63	1719.53	1719.34	1719.81	1719.65	1719.61	1719.68	1719.59	1719.77	1719.36
44	1715.72	1723.55	1717.35	1717.41	1717.41	1717.41	1717.12	1717.28	1717.40	1717.33	1717.44	1717.50	1717.40	1717.44	1717.35
58	1715.83	1724.16	1714.43	1714.50	1714.49	1714.49	1714.46	1714.43	1714.62	1714.50	1714.46	1714.57	1714.47	1714.61	1714.53
64	1715.88	1725.28	1717.29	1717.38	1717.37	1717.37	1717.28	1717.06	1717.57	1717.41	1717.33	1717.42	1717.33	1717.53	1717.10
94	1714.68	1723.90	1715.97	1716.04	1716.04	1716.04	1715.93	1716.00	1716.07	1715.98	1716.05	1716.12	1716.02	1716.09	1716.10
Sum absolute error		63.71	15.65	15.93	15.92	15.92	15.47	15.27	16.25	15.83	16.00	16.08	15.83	16.17	**15.10**
Sum absolute error (%)		3.71	0.91	0.93	0.93	0.93	0.90	0.89	0.95	0.92	0.93	0.94	0.92	0.94	**0.88**
		**S: SVR**		**A: ANN**		**L: LSTM**		**P: POA-ANN**							

**Fig. 8:**
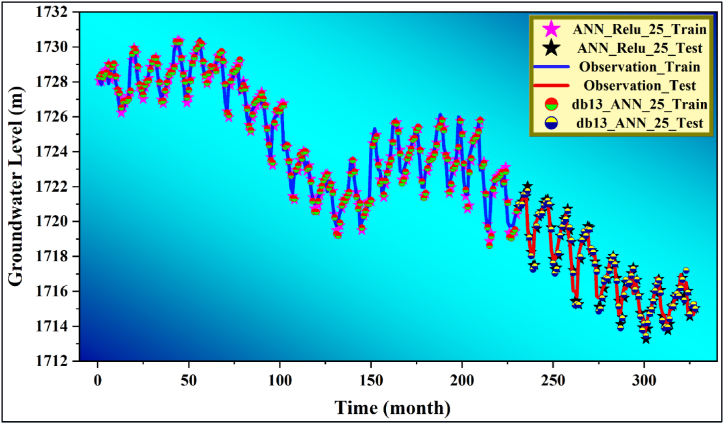
Compare the best model and pure model of it versus observation data.

In Fig. 8, the variation of the estimation results of the ANN_Relu_25 and db13_ANN_25 models with the actual data are temporally compared. Accordingly, both models showed satisfactory and promising results in estimating GWL (t+1) values. However, when the training and test time series are evaluated in detail, it can be deduced that the ANN_Relu_25 model shows the best results in terms of better overlapping with the real values.

**Fig. 9:**
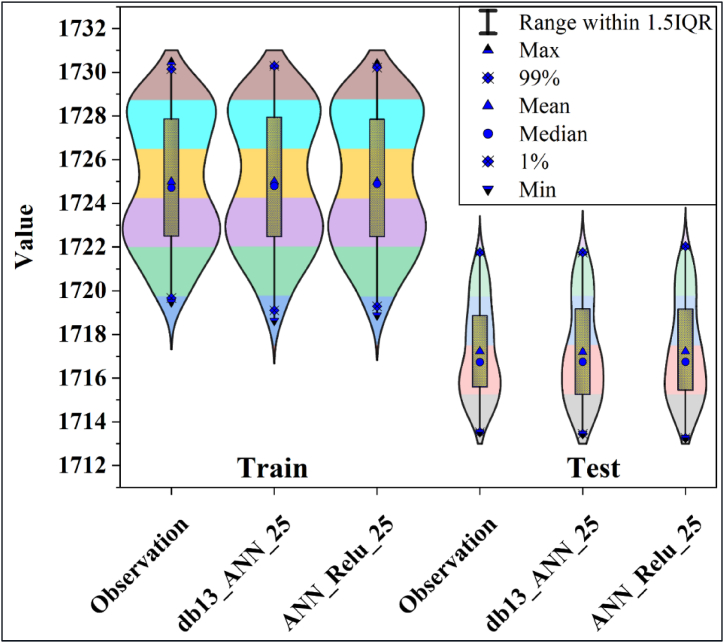
Violon plot of training and testing phase for the best model and its raw model.

Violin plots are shown in Fig. 9 to compare the prediction results of the ANN_Relu_25 and db13_ANN_25 models. These graphs evaluated the similarity of the max, min, median and percentile distributions between the actual and predicted values. Accordingly, it is noteworthy that the ANN_Relu_25 model represents the real data set slightly better than the db13_ANN_25 model. Therefore, it was revealed that the ANN_Relu_25 model showed the best estimation results.

**Fig. 10:**
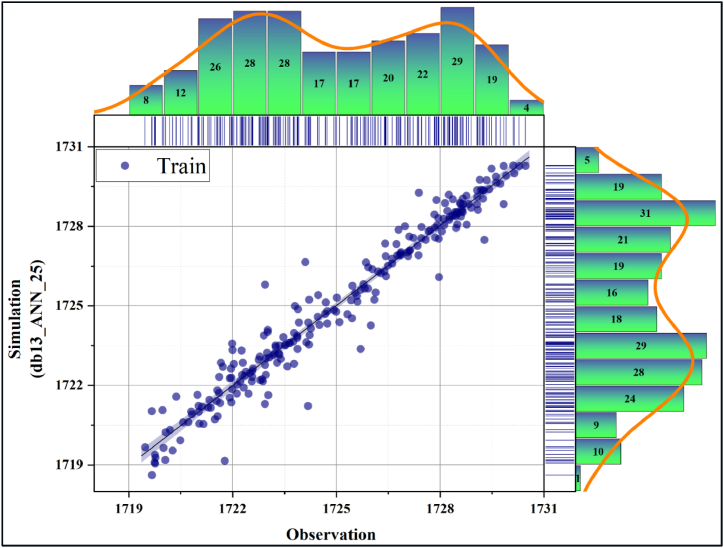
Scatter plot of the best model during the testing phase.

**Fig. 11:**
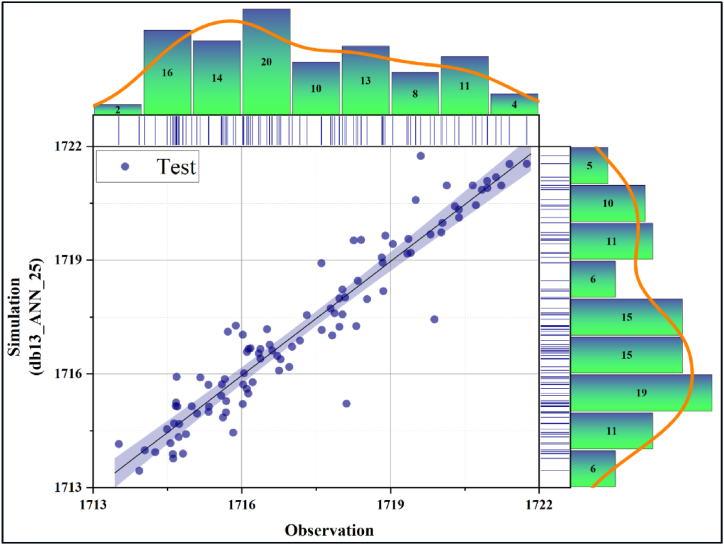
Scatter plot of the best model during the testing phase.

**Fig. 12:**
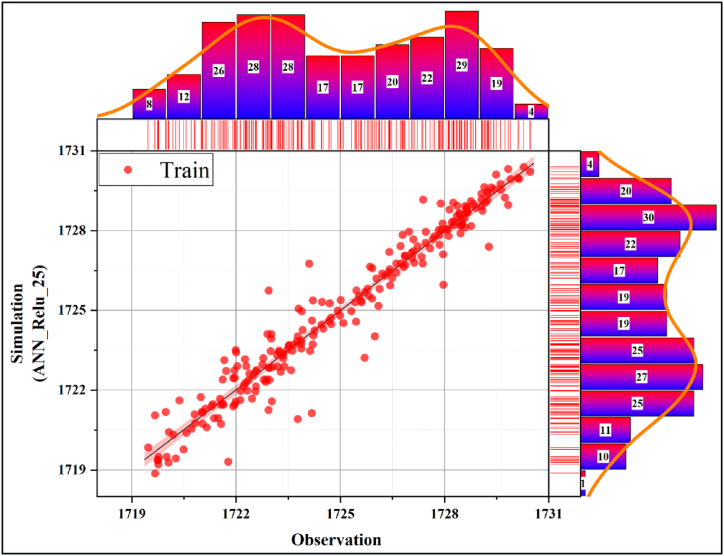
Scatter plot of the best raw model during the training phase.

**Fig. 13:**
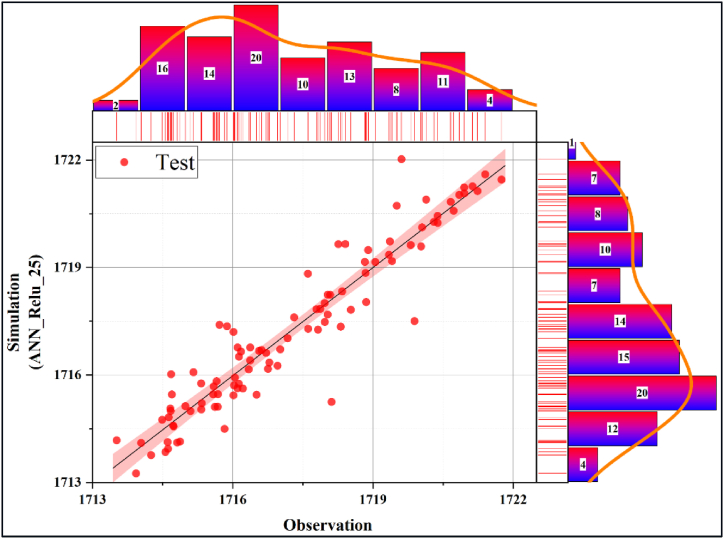
Scatter plot of the best raw model during the testing phase.

In Fig. 10, Fig. 11, the performance evaluation of the db13_ANN_25 model was made with the scatter diagram in the training and testing phases, respectively. Scatter diagrams fit a regression line to the data to reveal the relationship between the real time series and the predicted model. Models clustered near this line were interpreted as having high accuracy. Accordingly, it can be deduced that the db13_ANN_25 model has a very high estimation accuracy during the training and testing phases. In addition, it is seen that the training accuracy is slightly higher than the test accuracy. Fig. 12, Fig. 13 present the scatter diagrams of the ANN_Relu_25 model during the training and testing phases, respectively. Accordingly, it is seen that the estimated accuracy of the model in both stages is promisingly high.

**Fig. 14:**
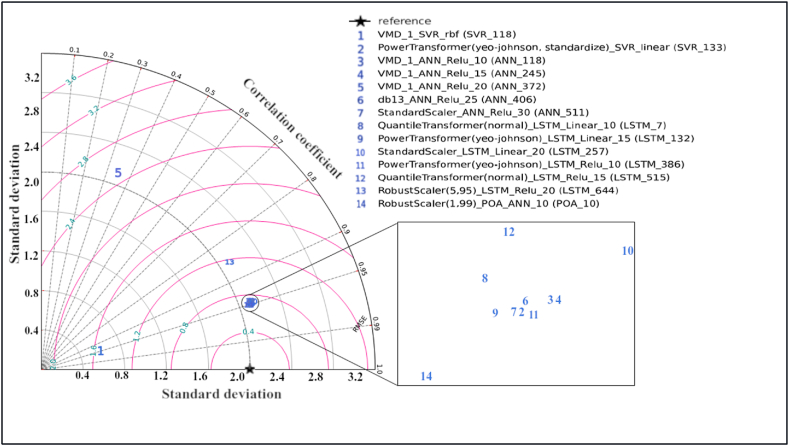
Taylor diagram of the models in the testing phase for best model of each model type.

In Fig. 14 Taylor diagrams are presented to present the relative analysis of the GWL estimates. These diagrams help to select the optimum model by comparing the relationship between the real and predicted time series, the error level and standard deviation values. Accordingly, models 10, 11, 14 and 34 stand out in prediction accuracy (R:0.95). When these models were evaluated according to their RMSE values, it was decided that model number 14 (Robust scaler - POA_ANN_10) was the highest with the lowest error.

### 3.2 Discussion

This study found that ANN and POA-ANN models showed the most accurate results in predicting GWL. Furthermore, it was revealed that the prediction accuracy of stand-alone ML and DL models can be improved by applying optimization, signal decomposition, and pre-processing data transformations. When the results were evaluated according to the lowest AIC values, the most accurate predictions were got with the ANN and POA-ANN models, while the weakest predictions were found with the SVM model. It has also been inferred that the EMD data decomposition approach improves GWL prediction accuracies compared to other preprocessing techniques based on low AIC values. For GWL estimation, it can be concluded that the Daubechies class is the most appropriate wavelet for the regime of the basin and the climate structure of the region. Decomposition methods (EMD and VMD) and wavelet transform pre-processing techniques generally showed more effective results in GWL estimation than Statistical methods (Table 4, Table 5). The findings of this study are consistent with Wu et al. [75], Lin et al. [76], Zhang and Zheng [48] and Panahi et al. [77]. VMD algorithm improved the performance of the ELM algorithm in predicting GWL in northwestern China [78]. Lin et al. [76] tested various combinations of LSTM, gated recurrent unit (GRU), and VMD techniques for GWL prediction in the Qoşaçay plain, Iran. The analysis results revealed that the VMD technique improved the performance of the DL technique. Zhang and Zheng [48] CEEMDAN, integrated the VMD data decomposition technique into a convolutional neural network gated repetitive unit prediction model (CNN-GRU). As a result, it was revealed that CEEMDAN and VMD models improved the prediction accuracy of the CNN-GRU model. Panahi et al. (2023) used ELM, VMD and Crow search algorithm (CSA) to estimate surface water TDS in Karoon River. The results showed that CSA and VMD techniques improved the prediction performance of the ELM model.

In this study, results revealed that various data decomposition techniques enhance the performance of machine learning models in GWL prediction. DWT achieves improved GWL prediction by decomposing input variables into sub bands at various frequencies, EMD determines the fundamental characteristics and variation modes of the inputs, and VMD extracts noise from the data, thereby enhancing the GWL prediction accuracy of ML models [59,79,80]. Adamowski and Chan [81] employed DWT in combination with ANN and integrated moving average (ARIMA) models to estimate groundwater level (GWL) at two sites in the Chateauguay basin in Quebec, Canada. The analysis revealed that the WA-ANN hybrid approach, which outperformed ANN and ARIMA models, provided more accurate GWL estimates. Moreover, the study found satisfactory results were achieved using monthly total precipitation, average temperature, and GWL data as inputs for GWL estimation. The study by Adamowski and Chan [81] supports the current study's findings. Wu et al. [75] applied a multi-step modeling framework called the WT-MLSTM hybrid model, which combines WT with a LSTM network to model groundwater level (GWL) values. It was identified that the integrated WT-MLSTM model outperformed LSTM, MLSTM, and WT-LSTM models. The analysis indicated that the WT technique enhances the performance of the LSTM algorithm. Additionally, it was stated that the WT-MLSTM model is more advantageous than the SVM algorithm. The study by Wu et al. [75] supports the present study's findings. Ebrahimi and Rajaee [82] integrated Multiple Linear Regression (MLR), ANN, and SVM models with DWT for predicting 1-month forward GWL values in the Qom plain, Iran. The analysis revealed that Meyer and Db5 wavelets yielded more accurate results than others. This finding contradicts the previous study mentioned. However, this discrepancy can be attributed to differences in the precipitation regime and data period between the regions studied.

When the errors of the GWL prediction value of the observation and first-order models were evaluated during the validation period (Table 6), it was concluded that the POA-ANN hybrid approach could effectively predict extreme values due to having a lower error percentage than other models. Additionally, many studies indicate that more precise extreme value predictions can be made if parameter optimization is performed with metaheuristic algorithms such as POA. The traditional heuristic approach provides insufficient convergence compared to meta-heuristic algorithms due to its poor convergence speed and the disadvantages of skipping global optimum and showing local optimum. Therefore, the study determined that optimization techniques such as PSO and GA produced promising outputs by optimizing ANN parameters [83,84]. Seifi et al. [85] various ML optimization algorithms with grasshopper optimization algorithm (GOA), cat swarm optimization (CSO), weed algorithm (WA), genetic algorithm (GA), krill algorithm (KA), and particle swarm optimization (PSO) optimized the parameters of the models. By estimating the GWL values of the established hybrid model file, it has been shown that the GOA-ANFIS hybrid approach can predict the GWL values at the highest level and the SVM model is the weakest. It has also been revealed that metaheuristic optimization techniques significantly improve GWL prediction accuracy. Dash et al. [86] indicated that the ANN-GA hybrid model best predicts GWL data. Kayhomayoon et al. [87] proved that the ant colony optimization for continuous domains (ACOR) optimization technique significantly improved the GWL prediction accuracy of the ANFIS model. Existing studies support that POA-based ML models improve model accuracy in estimating GWL and capturing extreme values more effectively.

Groundwater levels are based on many meteorological, hydrological and climatic variables. Chen and Hu [88] stated that groundwater level, soil moisture changes and surface evaporation have an important relationship. GWL variation is affected by climate variables and hydrological factors such as precipitation intensity, evaporation and transpiration, surface runoff and drainage [[89], [90], [91]]. Triki et al. [92] reported that temperature affects the change of the GWL more than rainfall. Dudley et al. [93] reported groundwater levels based on hydrologic and meteorological variables, especially streamflow and base flow, which are the most critical parameters. The study used precipitation, temperature evaporation and historical GWL data as input for GWL estimation. When the existing literature is examined, the parameters used in GWL estimation are very similar to the current study. But, GWL data varies according to many parameters. This situation constitutes the main difficulties and limitations in the precision prediction of GWL. In future studies, it is recommended to evaluate the performance by presenting a model with additional data, such as stream flow, evaporation, and pressure, to make more precise GWL predictions.

## 4 Conclusion

This study used precipitation, temperature and evaporation data and historical series GWL data to estimate one-month shifted GWL values in the Hamedan-Bahar Plain, Iran. ANN, SVM, LSTM and POA-ANN models were used as the basis for estimating GWL values. To evaluate the performance of these established models, various pre-processing techniques such as EMD, DWT, VMD and statistical methods were combined with these models. Basically, it is aimed to establish a hybrid structure of machine learning, deep learning and metaheuristic optimization techniques with data decomposition and normalization techniques in GWL prediction.

The accuracy of the established models was calculated based on various statistical and graphical metrics. According to the results, the ANN (db13_ANN_25, ANN_Relu_25) and POA-ANN models achieved better results in predicting endogenous GWL values. The results also indicate that the EMD data decomposition method outperforms other pre-processing techniques with lower AIC values. This study's findings are critical for managing drought-prone regions in Iran, irrigation planning, and developing climate change adaptation strategies. It has also been found that the estimation accuracy is generally increased by combining the stand-alone ML and DL models with data pre-processing, data decomposition and bio-inspired algorithms. In addition, as a result of the study, it was revealed that the performance of the established prediction models improved when scaled with the standardization and normalization of the data. When various signal processing techniques are combined with artificial intelligence techniques, it has emerged that the performance of artificial intelligence models in GWL estimation increased by decomposing noise in meteorological data and modeling different frequency structures.

The number of neurons has significant effects on the complexity of the model, learning capacity, generalization ability and over-fitting status. Increasing the number of neurons up to 30 in the training of The ANN model reached the highest levels in GWL prediction. The most accurate predictions were obtained by increasing the number of neurons to 30 and using the Relu activation function. Additionally, when the general performances of data preprocessing techniques were evaluated, it was determined that DWT and statistical preprocessing produced more accurate GWL estimates than VMD and EMD. This shows that DWT can parse input data into different frequency levels compared to EMD and VMD. In addition, it has been deduced that the db mother wavelet class allows producing more accurate predictions in GWL estimation than other wavelets. This situation coincides with the ability to define subcomponents of the db mother wavelet using a limited number of coefficients.

Combining more than one method in GWL estimation allows an approach that is limited on its own to be strengthened with various data processing techniques. Thus, GWL estimates can be made more precisely. In this context, the ability of decision makers, policy makers and stakeholders to take action in water management and environmental practices increases, enabling effective regional planning, energy and agricultural production. Additionally, precise GWL estimates help develop flood and drought management and climate change adaptation strategies.

In future studies, the combination of various ML and DL techniques with bioinspired optimizations such as Firefly Algorithm, Grasshopper Optimization Algorithm, Dragonfly Algorithm, Butterfly Optimization Algorithm, Whale Optimization Algorithm, and Grey Wolf Optimization, as well as data decomposition techniques such as Singular Spectrum Analysis, Seasonal Decomposition of Time Series, Local Regression, and Local Mean Decomposition, is recommended for predicting GWL values. In addition, in order to estimate more precise GWL values in future studies, sensitivity analysis can be made by including various parameters such as stream flow, aquifer parameters, evaporation, wind speed, relative humidity and pressure into the model, and the most effective parameters on GWL can be presented as input to the model.

## Funding

Open access funding provided by Lulea University of Technology. The work was supported by the 10.13039/501100003698KICT Research Program (project no. 20230166-001, Development of Coastal Groundwater Management Solution) funded by the Ministry of Science and ICT.

## Availability of data and material

They are available from the first author on reasonable request.

## Ethics approval

Not applicable.

## Consent to participate

All the authors mentioned in the manuscript have agreed for authorship, read and approved the manuscript.

## Consent for publication

All the authors mentioned in the manuscript have given consent for submission and subsequent publication of the manuscript.

## Code availability

Not applicable.

## CRediT authorship contribution statement

**Mohsen Saroughi:** Writing – original draft, Visualization, Software, Formal analysis, Data curation, Conceptualization. **Ehsan Mirzania:** Writing – original draft, Visualization, Formal analysis, Data curation. **Mohammed Achite:** Writing – original draft. **Okan Mert Katipoğlu:** Writing – review & editing, Writing – original draft, Investigation. **Nadhir Al-Ansari:** Writing – review & editing, Writing – original draft, Visualization, Validation, Supervision. **Dinesh Kumar Vishwakarma:** Writing – review & editing, Writing – original draft, Visualization, Validation, Supervision. **Il-Moon Chung:** Writing – review & editing. **Maha Awjan Alreshidi:** Writing – review & editing. **Krishna Kumar Yadav:** Writing – review & editing.

## Declaration of competing interest

The authors declare that they have no known competing financial interests or personal relationships that could have appeared to influence the work reported in this paper.
